# Choosing the appropriate funding sources; lesson learned from Maternal Neonatal Child Health evidence-based planning in 3 districts in Papua, Indonesia

**DOI:** 10.1186/1471-2458-14-S1-O19

**Published:** 2014-01-29

**Authors:** Muhamad Faozi Kurniawan, Tiara Marthias, Deni Harbianto, Digna Purwaningrum, Likke Putri

**Affiliations:** 1 CHPM Faculty of Medicine, Gadjah Mada University, Yogyakarta 55281, Indonesia

## Background

Special Autonomy policy grants greater authority for Papua government to manage, organise and finance the province in improving social economic development. Papua province received Special Autonomy status along with other provinces; West Papua and Aceh. As part of that policy, Papua receives special funding from the central government called Special Autonomous Fund (Dana OTSUS). OTSUS fund provides the government more authority for planning and budget allocation. The objective of the present study was to describe the programmatic planning impact of evidence-based approach on Maternal, Newborn and Child Health programme in 3 districts in Papua province. It was also to analyse the challenges in choosing appropriate funds to finance the MNCH programme.

## Materials and methods

Descriptive study, quantitative data pooling and measurements based on planning documents were employed. This study analysed, described and observed the impact of OTSUS budget allocation process and other funds in Papua, with the context of evidence-based approach implementation. Evidence-based approach is an improved planning method comprising of identifying local health problems, choosing appropriate strategies, programme planning, budgeting and financing.

## Results

From 2011 to 2013, there was a shift in funding sources allocation in three Papua districts. OTSUS fund has been utilised as the major funding source. OTSUS fund had increased during the last 3 years. However, other funds had been reduced, such as Local Revenue fund. Consequently, it caused a decrease in preventive and promotive programmes, which were funded by Local Revenue and General Allocation Funds. Historically, the planning process had not been based on specific local problems. This challenge affected the choice of appropriate funding scheme. Local Government’s main concern was only at the fund disbursement timeliness, not on the actual strategies aimed to solve the problems.

## Conclusions

There is a need for scaled up use of evidence-based planning, particularly for improving fund allocation for MNCH programmes. The government should also increase its funding allocation for MNCH in general.

